# Comparison of intravenous vs intravenous with step-down to oral antibiotic treatment course for Streptococcus and Enterococcus bloodstream infections

**DOI:** 10.1017/ash.2025.168

**Published:** 2025-05-19

**Authors:** Kelsey Bouwman, Jacob W. Pierce, Jennifer Emberger, Alexandra Te Stang, Paul Vos, Aaron M. Kipp, Nicole C. Nicolsen

**Affiliations:** 1 Department of Pharmacy, East Carolina University Health Medical Center, Greenville, NC, USA; 2 Division of Infectious Diseases, Department of Internal Medicine, Brody School of Medicine at East Carolina University, Greenville, NC, USA; 3 Department of Public Health, Brody School of Medicine at East Carolina University, Greenville, NC, USA

## Abstract

**Objective:**

To compare clinical failure of intravenous vs intravenous with oral step-down antibiotic treatment for Streptococcus and Enterococcus bloodstream infection.

**Design and setting:**

Multicenter, retrospective, cohort study at one academic medical center and eight community hospitals.

**Patients:**

Hospitalized adult patients with blood cultures positive for Streptococcus or Enterococcus were included. Patients were excluded if they had complicated infection, had polymicrobial bacteremia, received less than 5 days of therapy, or died before completing therapy.

**Methods:**

Patients who completed intravenous therapy were compared with patients who transitioned to oral therapy after 3 to 7 days. The primary endpoint was clinical failure, defined as 90-day all-cause mortality or recurrent bacteremia. The primary analysis excluded patients with unknown outcomes, and the sensitivity analysis treated them as failures.

**Results:**

429 patients were included (intravenous group: n = 225; oral step-down group; n = 204). The intravenous group had more comorbidities and vasopressor use. The intravenous group had a higher risk of clinical failure in the primary analysis (17.5% vs. 8.8%; adjusted OR 2.14 [95% CI, 1.09–4.2]; *p* = 0.03) while the sensitivity analysis found no difference in clinical failure (adjusted OR 1.1 [95% CI, 0.69–1.74], *p* = 0.69). The oral step-down group had a mean length of stay of 9.2 days shorter than the intravenous group ([95% CI, 7.5–11.0]; *p*<0.001).

**Conclusion:**

Oral step-down therapy was not associated with an increased risk of clinical failure compared to a full course of intravenous therapy for uncomplicated Streptococcus and Enterococcus bloodstream infections. Patients with more comorbidities or who required vasopressors were less likely to be switched to oral therapy.

## Background

Historically, the standard approach for treatment of bloodstream infections (BSI) was intravenous (IV) antibiotics for the entire duration of treatment. However, there are several well-known disadvantages of prolonged IV therapy including increased costs, line-associated complications, and increased hospital length of stay (LOS).^1,2^ Many providers are gaining comfort with transition to oral therapy for bacteremia. For Gram-negative BSI, there is a growing body of evidence showing that oral step-down (PO-SD) therapy has beneficial outcomes leading to reduced hospital LOS and line-associated complications, with no increase in mortality.^3–5^ Therefore, PO-SD therapy has become a more widely accepted practice in Gram-negative BSI.

There are less data available to support PO-SD therapy for Gram-positive BSI, but retrospective evidence is starting to indicate favorable outcomes with PO-SD therapy in clinically stable patients with uncomplicated Streptococcus BSI.^6–13^ However, the clinical outcomes data are limited mostly to small retrospective reviews that were variable in terms of organism treated, severity of illness, agents selected, and doses utilized. Limited studies have looked at clinical outcomes of PO-SD therapy for BSI with Enterococcus, and studies that included Enterococcus generally had fewer cases of BSI due to this organism.^7,8,13–15^ The objective of this study is to compare clinical failure of IV vs IV with PO-SD antibiotic treatment for Streptococcus and Enterococcus BSI.

## Methods

### Study design

This was a single-system, multicenter, retrospective cohort study conducted across nine hospitals in the East Carolina University Health (ECUH) system. ECUH includes 1,708 beds across an academic medical center with two campuses and seven community hospitals. Hospitalized patients at least 18 years of age, who started active antibiotics within 48 hours of blood cultures positive for Streptococcus or Enterococcus from November 2019 to October 2023, were identified for screening. Patients were excluded if they had complicated infections (defined below); persistent bacteremia; obvious lack of source control (e.g., intra-abdominal infection without drainage); polymicrobial bacteremia; received less than five days of active antibiotics to account for contaminants; died, transitioned to hospice, or transferred to a non-ECUH facility before completing therapy; switched back to IV therapy for nothing by mouth (NPO) status; or had an unclear antibiotic end of therapy (defined below). Patients who completed a full course of IV therapy were compared with patients who transitioned from IV to PO-SD therapy after at least 3 days but no more than 7 days of IV therapy.

### Endpoints

The primary endpoint was a composite outcome of clinical failure defined as 90-day all-cause mortality or 90-day recurrent bacteremia with the same organism as the initial episode. Secondary endpoints included 90-day all-cause mortality, 90-day recurrent bacteremia, 90-day all-cause readmission, and hospital LOS.

### Definitions

Recurrent bacteremia was defined as any positive blood culture with the same organism one or more days after completing antibiotic therapy. Source was determined by the treating physician based on documentation from retrospective chart review, and it was listed as unknown if source was not clearly documented. Complicated infection was defined as meningitis, osteomyelitis, septic joint, endocarditis, or catheter-associated bacteremia without catheter removal or replacement. Persistent bacteremia was defined as positive blood cultures after four days of active therapy, if repeat blood cultures were obtained. Patients were considered to have an unclear antibiotic end of therapy if they received dalbavancin or were started on prophylactic antibiotics (for any indication) after finishing treatment.

Patients were considered to be at high risk of clinical failure if they met one of the following criteria: central venous catheter present at time of bacteremia that was replaced; line that was retained but not thought to be the source; cardiac device; prosthetic joint or heart valve; other foreign material (e.g., ventriculoperitoneal shunt, abdominal aortic aneurysm repair, orthopedic fixation hardware); history of human immunodeficiency virus, bone marrow transplant, IV drug use (IVDU); or on immunosuppressive medications (e.g., methotrexate, mycophenolate, azathioprine, leflunomide, steroid use equivalent to prednisone 20 mg/day or greater for at least 30 days, chemotherapy or biologic within the last 21 days).

Duration of therapy was defined as total days of therapy from day 1 of active antibiotics. Oral antibiotics were categorized as highly bioavailable if their bioavailability was >90%. Oral antibiotics with high bioavailability included fluoroquinolones, linezolid, clindamycin, trimethoprim-sulfamethoxazole, high-dose amoxicillin (HD-amoxicillin), and cephalexin. Oral antibiotics with lower bioavailability included amoxicillin-clavulanate, low-dose amoxicillin (LD-amoxicillin), penicillin V potassium, cefdinir, cefadroxil, and cefpodoxime. HD-amoxicillin was defined as a dose of 1000 mg three times per day and could be adjusted for renal function, and LD-amoxicillin was defined as any dose lower than that. All antibiotic doses were assessed for appropriateness based on ECUH dosing policies, taking into account indication and patient-specific factors such as weight and renal function.

### Microbiology studies

Bacterial identification and antimicrobial susceptibility testing varied slightly based on hospital site. In general, the health system utilizes biochemical testing, MicroScan WalkAway, and Vitek® MS (current library 3.0) which uses Matrix Assisted Laser Desorption Ionization (MALDI-TOF) technology. Susceptibility results were generally interpreted according to the Clinical and Laboratory Standards Institute recommendations in place at that time.

### Statistical analysis

Assuming 25% clinical failure in each group, the *Z*-test with continuity correction for the difference of two proportions showed that 252 patients in each group would provide 80% power to show non-inferiority with a 10% margin as is often done.^9,13,15^ Chi square test was used for categorical variables and *t*-test for continuous variables. A multivariable logistic regression was completed to adjust for confounders between groups including organism, Charlson Comorbidity Index (CCI), vasopressor use, high risk of clinical failure, and duration of therapy. These variables were chosen because they had larger differences between groups or because they have been shown in previous data to make a difference in clinical outcomes. 11.1% of patients in the IV group and 16.2% of patients in the PO-SD group had unknown outcomes for the primary endpoint, so a primary and sensitivity analysis were completed. The primary analysis excluded patients with unknown outcomes, and the sensitivity analysis treated them as failures. A *P*-value ≤0.05 was considered to be statistically significant. All statistical tests were performed via Statistical Analysis System (SAS^®^) Institute Inc, Cary, NC software.

## Results

**Figure 1.**
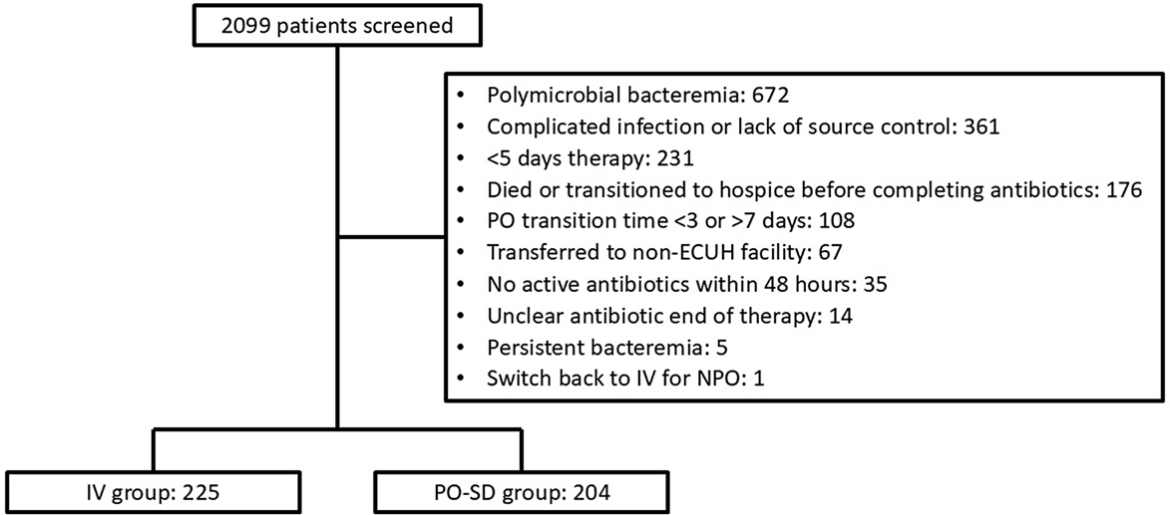
Patient selection. *Note*:. “PO-SD”, oral step-down; “IV”, intravenous; “ECUH”, East Carolina University Health; “NPO”, nothing by mouth.

A total of 2,099 patients were screened. The most common reasons for exclusions were polymicrobial bacteremia, complicated infections, and receiving less than five days of active antibiotics (Figure 1). This resulted in a total of 225 patients in the IV population and 204 patients in the PO-SD population.

Table 1 describes baseline characteristics, which were comparable between groups in terms of age, gender, weight, race, and high risk of clinical failure. The patients in the PO-SD group had a lower CCI and fewer patients who required vasopressors. About half of the patients in each group had an infectious diseases (ID) or antimicrobial stewardship program (ASP) consult. Blood culture clearance was confirmed in most patients. Overall, Enterococcus (18.9%) made up much less of the full patient population than Streptococcus, and less patients with Enterococcus were in the PO-SD group (15.2% vs 22.2%). The most common Streptococcus species were *Streptococcus pneumoniae* and viridans group Streptococci. When source was known, it was most commonly attributed to a skin and soft tissue infection or pneumonia.

**Table 1.** Baseline characteristics

	IV (n = 225)	PO-SD ( 204)	*p*-value
**Male; n (%)**	121 (53.8%)	96 (47.1%)	
**Age (years); mean (SD)**	65.1 (±15.2)	63.7 (±16.9)	0.34
**Weight (kg); mean (SD)**	91.6 (±31.1)	88.7 (±29.2)	0.36
**Race; n (%)**			
Caucasian	127 (56.4%)	123 (60.3%)	
African American	89 (39.6%)	71 (34.8%)	
Other	9 (4%)	10 (4.9%)	
**High risk of clinical failure; n (%)**	81 (36%)	66 (32.4%)	0.66
**Duration of therapy; median (range)**	14 (5–20)	13 (6–20)	
**ID or ASP consult; n (%)**	112 (49.8%)	118 (57.8%)	0.09
**Large academic medical center; n (%)**	146 (64.9%)	105 (51.5%)	0.005
**Charlson comorbidity index; mean (SD)**	4.27 (±2.3)	3.76 (±2.3)	0.02
**Vasopressor required; n (%)**	54 (24.0%)	15 (7.4%)	<0.001
**Repeat blood culture obtained; n (%)**	202 (89.8%)	156 (76.5%)	<0.001
Days to blood culture clearance; mean (SD)	2.9 (±2.9)	2.1 (±1.2)	<0.001
**Echocardiogram obtained; n (%)**	169 (75.1%)	116 (56.9%)	<0.001
**Source; n (%)**			
Unknown	73 (32.4%)	51 (25%)	0.08
Skin	44 (19.6%)	53 (26%)	0.05
Pneumonia	37 (16.4%)	51 (25%)	0.02
Gastrointestinal	26 (11.6%)	20 (9.8%)	0.78
Genitourinary	23 (10.2%)	17 (8.3%)	0.78
Oropharyngeal	10 (4.4%)	12 (5.9%)	0.25
Catheter	12 (5.3%)	0 (0%)	–
**Organism; n (%)**			
*Enterococcus* spp.	50 (22.2%)	31 (15.2%)	0.07
*Streptococcus* spp.	175 (77.8%)	173 (84.8%)	
Group A *Streptococcus*	18 (8.0%)	27 (13.2%)	0.07
*Streptococcus pneumoniae*	22 (9.8%)	36 (17.6%)	0.02
Viridans group Streptococci	45 (20%)	41 (20.1%)	0.17
*Streptococcus anginosus*	28 (12.4%)	12 (5.9%)	0.01
Other *Streptococcus*spp.	62 (27.6%)	57 (27.9%)	0.9

Note. IV, intravenous; PO-SD, oral step-down; SD, standard deviation; kg, kilograms; ID, infectious disease; ASP, antimicrobial stewardship program; spp; species.

Median duration of therapy was 14 (5–20) days in the IV group and 13 (6–20) days in the PO-SD group (Table 1). The most common IV antibiotics utilized as targeted therapy in the total patient population were ceftriaxone (56.6%), ampicillin (10.5%), and piperacillin-tazobactam (9.3%). Overall, 98.1% of IV antibiotics were determined to be dosed appropriately. The mean days to switch to oral antibiotic therapy was 4.75 days, and the most common oral antibiotics utilized were HD-amoxicillin (21.6%), amoxicillin-clavulanate (21.1%), LD-amoxicillin (14.2%), and linezolid (12.7%). Overall, 98.5% of PO antibiotics were determined to be dosed appropriately. The majority (79.1%) of patients who received amoxicillin-clavulanate were dosed at 875–125 mg twice daily. In total, 52.5% of oral antibiotics utilized were classified as highly bioavailable and 47.5% were classified as having lower bioavailability. A full list of antibiotic selection is described in Table 2.

**Table 2.** Antibiotic selection

	IV (n = 225)	PO-SD (n = 204)
**Targeted (IV) antibiotic; n (%)**		
Ceftriaxone	118 (52.4%)	125 (61.3%)
Ampicillin	23 (10.2%)	22 (10.8%)
Piperacillin-tazobactam	24 (10.7%)	16 (7.8%)
Vancomycin	19 (8.4%)	11 (5.4%)
Penicillin G	14 (6.2%)	12 (5.9%)
Cefazolin	8 (3.6%)	4 (2.0%)
Ampicillin-sulbactam	5 (2.2%)	7 (3.4%)
Cefepime	4 (1.8%)	5 (2.5%)
Moxifloxacin	2 (0.9%)	2 (1.0%)
Ertapenem	3 (1.3%)	0
Daptomycin	3 (1.3%)	0
Meropenem	2 (0.9%)	0
**PO-SD antibiotic; n (%)**		
HD-amoxicillin		44 (21.6%)
Amoxicillin-clavulanate		43 (21.1%)
LD-amoxicillin		29 (14.2%)
Linezolid		26 (12.7%)
Levofloxacin		19 (9.3%)
Moxifloxacin		15 (7.4%)
Cephalexin		13 (6.4%)
Cefuroxime		6 (2.9%)
Cefdinir		5 (2.5%)
Clindamycin		3 (1.5%)
Penicillin V potassium		1 (0.5%)
**PO-SD bioavailability**		
High		107 (52.5%)
Low		97 (47.5%)

Note. IV, intravenous; PO-SD, oral step-down; HD, high dose; LD, low dose.

For the primary endpoint, the IV group had a significantly higher risk of clinical failure than the PO-SD group in the primary analysis (17.5% vs. 8.8%; OR 2.2 [95% CI, 1.16–4.20]; *p* = 0.02), while there was no difference in the sensitivity analysis (26.7% vs. 23.5%; OR 1.18 [95% CI, 0.76–1.83]; *p* = 0.46) (Table 3). Results did not change substantially following adjustment for potential confounders for the primary analysis (adjusted OR 2.14 [95% CI, 1.1–4.2]; *p* = 0.03) and the sensitivity analysis (adjusted OR 1.1 [95% CI, 0.7–1.7]; *p* = 0.69). Nevertheless, the lower 95% confidence interval indicates we cannot rule out the possibility that the IV group has 30% lower risk of clinical failure. The difference in clinical failure was primarily driven by 90-day mortality with the IV group having a higher risk of 90-day mortality in the primary analysis (adjusted OR 2.3 [95% CI, 1.07–4.9]; *p* = 0.03) and no difference in the sensitivity analysis (adjusted OR 1.03 [95% CI, 0.64–1.67]; *p* = 0.89). Mean LOS was significantly shorter in the PO-SD group (5.1 days vs 14.3 days; *p* < 0.001). Choice of oral antibiotics did not statistically impact the primary endpoint of clinical failure, though the primary outcome was numerically lower in the highly bioavailable group in the primary analysis (7.8% vs 11.1%; *p* = 0.4). Table 3 presents the full results for the unadjusted and adjusted primary and secondary endpoints.

**Table 3.** Crude and adjusted primary and secondary endpoints comparing IV to PO-SD

Dichotomous endpoints	IV, n (%)	PO-SD, n (%)	Crude OR (95% CI)	*p*-value	Adjusted OR (95% CI)	*p*-value
**Clinical failure**						
Primary	35/200 (17.5%)	15/171 (8.8%)	2.2 (1.16–4.20)	0.02	2.14 (1.09–4.2)	0.03
Sensitivity	60/225 (26.7%)	48/204 (23.5%)	1.18 (0.76–1.83)	0.46	1.1 (0.69–1.74)	0.69
**90-day mortality**						
Primary	28/200 (14.0%)	11/171 (6.4%)	2.37 (1.14–4.9)	0.02	2.30 (1.07–4.9)	0.03
Sensitivity	53/225 (23.6%)	44/204 (21.6%)	1.12 (0.71–1.76)	0.62	1.03 (0.64–1.67)	0.89
**90-day recurrence**						
Primary	8/200 (4.0%)	6/171 (3.5%)	1.15 (0.39–3.37)	0.80	1.09 (0.36–3.4)	0.88
Sensitivity	33/225 (14.7%)	39/204 (19.1%)	0.73 (0.44–1.3)	0.22	0.70 (0.41–1.20)	0.19
**90-day readmission**						
Primary	92/203 (45.3%)	65/175 (37.1%)	1.4 (0.03–2.2)	0.11	1.51 (0.97–2.33)	0.06
Sensitivity	114/225 (50.7%)	94/204 (46.1%)	1.2 (0.82–1.80)	0.34	1.23 (0.83–1.84)	0.31
**Continuous endpoint**	**IV, Mean (SD)**	**PO-SD, Mean (SD)**	**Crude beta** **coefficient (95% CI)**	***p*** **-value**	**Adjusted beta coefficient (95% CI)**	***p*** **-value**
**Length of stay (days)**	14.3 (12.5)	5.1 (2.4)	2.16 (2.10–2.61)	<0.001	2.16 (1.93–2.41)	<0.001

Note. IV, intravenous; PO-SD, oral step-down; OR, odds ratio; CI, confidence interval.

## Discussion

In this multicenter, retrospective study of uncomplicated Streptococcus and Enterococcus bacteremia, PO-SD therapy was not associated with an increased risk of clinical failure compared to a full course of IV antibiotics. The length of stay was shorter in patients who received PO-SD, and 90-day recurrent bacteremia was low in both groups. While this study could not definitively demonstrate non-inferiority, it does add to the growing literature suggesting that evaluation of PO-SD in a randomized clinical trial is warranted.

There have been a few other smaller, single-center retrospective chart reviews evaluating outcomes in uncomplicated Streptococcus BSI that had similar results to our study. One study found that patients in the PO-SD group had lower risk of 30-day clinical failure compared to (IV) therapy (0% vs. 19.1%; *p* = 0.001) when treated with amoxicillin-clavulanate 875–125 mg twice a day.^10^ A second study found no difference in 30-day all-cause mortality (1% PO-SD vs. 4.1% IV; *p* = 0.25) with a variety of oral regimens used, primarily fluoroquinolones.^11^ A third study found no difference in 90-day clinical failure (18% PO-SD vs. 24.2% IV; *p* = 0.23) with a variety of oral regimens used, primarily cefdinir or amoxicillin.^12^ For Enterococcus, one systematic review found no difference in mortality between daptomycin and linezolid for vancomycin-resistant Enterococcus BSI.^14^ Additionally, a small subset of patients in another study received combination oral antibiotics for Enterococcus endocarditis and found no difference in their composite outcome for PO-SD.^15^ Finally, one study evaluated outcomes in uncomplicated Streptococcus and Enterococcus BSI together.^13^ They found PO-SD therapy was non-inferior to IV therapy for the primary outcome of clinical failure (9% vs 14%; *p* < 0.001), and this was driven by 90-day mortality difference (9% vs 12%). Isolates in both this study and our study were primarily Streptococcus, with a smaller subset of Enterococcus.

In contrast to Gandhi et al., where oral regimens were mostly fluoroquinolones, patients in our study primarily received oral beta-lactams for PO-SD therapy, including amoxicillin and amoxicillin-clavulanate. While there was not a statistically significant difference in clinical failure with use of agents with lower bioavailability compared to those with higher bioavailability, it is possible a larger number of patients could have shown a difference. With the long list of well-known adverse effects associated with fluoroquinolones, it is encouraging that our study describes positive outcomes with a large amount of beta-lactam usage and helps suggest dosing regimens that were successful. Total duration of therapy in our study (a median of 14 days in the IV group and 13 days in the PO-SD group) was similar to Gandhi et al. With the increase in literature supporting shorter durations of antimicrobial therapy in Gram-negative BSI, it is interesting to see that longer durations of therapy are still being used commonly in Gram-positive BSI.^16,17^ Duration of therapy for Streptococcus and Enterococcus BSI should be studied in more depth.

Our study applied a number of exclusion criteria in order to best isolate the association between PO-SD and clinical outcomes in uncomplicated BSI. This may limit the generalizability of our findings. Excluding patients with more severe or uncontrolled infections aimed to focus on antibiotic selection impacting outcomes rather than diagnosis. Excluding patients who received less than 5 days of active antibiotics attempted to account for contaminants, though it is not always clear with some of these specific organisms when they are contaminants vs pathogens. Excluding patients who died before completing therapy was done because it was likely that more than just antibiotic route was playing a role in the outcome. This did make up a large portion of the excluded patients (n = 176); however, all but two of these patients were not transitioned to oral therapy. Therefore, this should not have impacted the mortality in the PO-SD group. Additionally, we chose to allow patients to receive 3–7 days of IV antibiotics prior to transition to PO-SD therapy. While it is possible that 7 days could be considered a full course of therapy, the longer durations of therapy utilized in this study, and other similar studies, would suggest that most providers would not be comfortable with considering 7 days a full course.

The retrospective nature of this study does induce the potential for selection bias. As the baseline characteristics show, patients with higher CCI and vasopressor use were less likely to be transitioned to PO therapy. While there is potential for additional confounding factors, outcomes did not differ when adjusted for the variables discussed above, including CCI and vasopressor use. Nevertheless, only a randomized clinical trial can fully address concerns about confounding. The retrospective nature combined with data being limited to a single health system also led to the limitation of being unable to confirm the outcomes for certain endpoints. To combat this limitation, when outcomes could not be confirmed, we conservatively classified these endpoints as failures in the sensitivity analysis or excluded them from the primary analysis. Given that this was done as a worse-case scenario, it is possible that many of our unknown outcomes could have been successes. Finally, our study may have been underpowered to detect non-inferiority of PO-SD given the smaller sample size than what was suggested by our initial power calculation.

In conclusion, this study adds to the literature supporting that PO-SD antibiotic therapy likely does not increase risk of clinical failure compared to a full course of IV antibiotics for uncomplicated Streptococcus and Enterococcus BSI. Patients with higher CCI or who required vasopressors were less likely to be switched to oral therapy, and this should be taken into consideration when applying these results to clinical practice. Additionally, the lower number of patients with Enterococcus may impact clinical applicability for this organism. This study also supports the use of oral beta-lactams as options for PO-SD therapy for this indication, especially those with high oral bioavailability such as HD-amoxicillin.

## Research transparency and reproducibility

Contact corresponding author.
